# Single-port robotic-assisted radical prostatectomy: evaluating the Da Vinci SP system in minimally invasive urologic oncology

**DOI:** 10.1007/s00345-025-06018-0

**Published:** 2025-11-21

**Authors:** Kirolos Eskandar

**Affiliations:** Faculty of Medicine and Surgery, Helwan University, Cairo, Egypt

**Keywords:** Single-Port robotic surgery, Da vinci SP system, Radical prostatectomy, Minimally invasive urologic oncology, Prostate cancer surgery

## Abstract

Prostate cancer remains the most commonly diagnosed non-cutaneous malignancy among men worldwide, with over 1.4 million new cases annually. As surgical treatment evolves toward less invasive and more precise modalities, the emergence of the da Vinci Single-Port (SP) robotic system has marked a significant milestone in urologic oncology. Single-port robot-assisted radical prostatectomy (SP-RARP) aims to further minimize surgical trauma, enhance cosmesis, and expedite recovery—while preserving oncologic and functional outcomes. This review synthesizes the current literature on SP-RARP, detailing the technological advancements of the da Vinci SP system, surgical technique refinements, perioperative metrics, oncologic and functional results, and comparative outcomes versus multiport and conventional approaches. We also address the procedural limitations, the learning curve, and future directions, offering a comprehensive insight into the potential of single-port robotics in transforming prostate cancer surgery.

## Introduction

​Prostate cancer (PCa) is the most frequently diagnosed cancer among men in over half of the world’s countries, with an estimated 1.4 million new cases in 2020 [1]. In England, PCa has recently surpassed breast cancer as the most commonly diagnosed malignancy, with 55,033 new cases reported in 2023—a 25% increase over five years [2]. This rise is attributed to heightened awareness, aging populations, and improved diagnostic methods. Despite advancements, disparities persist; for instance, Black men face a 1.7-fold higher incidence and a 2.1-fold higher mortality rate compared to White men in the U.S., highlighting the need for equitable screening and treatment strategies [3].​

Radical prostatectomy (RP) has long been a cornerstone in the management of localized PCa. Initially performed via open perineal or retropubic approaches, RP has evolved significantly with the advent of minimally invasive techniques. Laparoscopic RP introduced in the 1990s offered reduced morbidity but was technically demanding [4]. The introduction of robot-assisted RP (RARP) in the early 2000s revolutionized the field by enhancing precision, reducing blood loss, and shortening recovery times [5]. Innovations such as the Retzius-sparing approach and nerve-sparing techniques have further improved functional outcomes, particularly concerning urinary continence and sexual function [6].​

The da Vinci Single-Port (SP) system represents the latest advancement in robotic surgery, aiming to minimize surgical trauma by utilizing a single incision. Early studies suggest that SP-RARP is feasible and safe, with potential benefits including reduced postoperative pain, shorter hospital stays, and improved cosmetic outcomes [7, 8]. However, challenges such as a steep learning curve and limited instrument triangulation remain.​.

This review aims to evaluate the current literature on SP-RARP, focusing on technological advancements, surgical techniques, perioperative outcomes, oncologic and functional results, and comparisons with multiport and traditional approaches. By synthesizing recent findings, we seek to provide a comprehensive understanding of the role of the da Vinci SP system in minimally invasive urologic oncology.

## Methodology

### Search strategy

This literature review was conducted using a structured and transparent approach to ensure comprehensive coverage of relevant studies. The review adhered to principles inspired by PRISMA (Preferred Reporting Items for Systematic Reviews and Meta-Analyses) to promote clarity and reproducibility. This review was prospectively registered with PROSPERO (PROSPERO 2025 CRD420251085012. Available from https://www.crd.york.ac.uk/PROSPERO/view/CRD420251085012).

Extensive searches were carried out across multiple scientific databases, including PubMed, Scopus, Web of Science, and Google Scholar, covering publications from January 2018 to March 2025. Search terms were formulated using a combination of keywords and medical subject headings (MeSH), such as: *“Single-Port Prostatectomy*,*” “da Vinci SP*,*” “SP-RARP*,*” “robotic-assisted radical prostatectomy*,*” “urologic oncology*,*”* and *“minimally invasive prostate surgery.”* Boolean operators (AND, OR) were applied to broaden and refine the results. Only articles published in English in peer-reviewed journals were considered.

### Study selection

To ensure relevance and quality, the following inclusion criteria were applied:

- *Language*: English only.
- *Content*: Studies directly discussing SP-RARP, particularly involving the da Vinci SP robotic platform.
- *Type of Publication*: Original research articles, systematic reviews, narrative reviews, and comparative studies.
- *Date Range*: Studies published between 2018 and 2025.

Articles were excluded if they met any of the following:

1. Non-peer-reviewed sources, conference posters, editorials, or opinion pieces.
2. Studies not specifically involving the da Vinci SP system.
3. Reports with unclear methods, insufficient detail, or inconclusive data.
4. Duplicated results or preliminary data without final peer-reviewed publication.

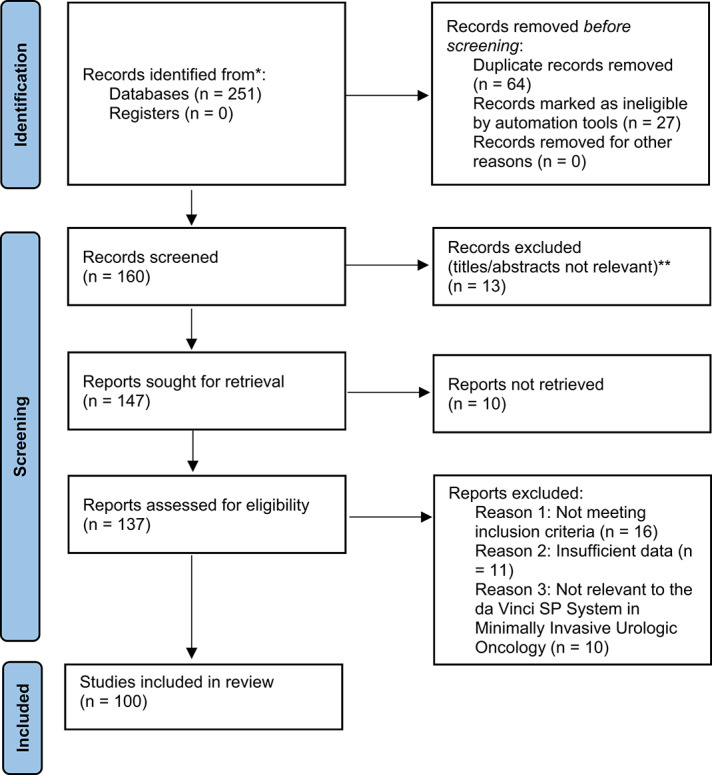


### Screening procedure

An initial pool of 251 records was retrieved. After duplicate removal using EndNote and manual verification, 160 unique articles remained. The screening process occurred in two phases:

1. *Title and Abstract Review*: Preliminary screening to eliminate irrelevant or ineligible studies.
2. *Full-Text Review*: Detailed evaluation of remaining articles to determine final eligibility based on inclusion/exclusion criteria.

Discrepancies between reviewers were resolved by discussion, with a third reviewer involved in cases of disagreement. The finalized selection comprised 99 articles.

### Data extraction

Key data were systematically extracted into a structured template, including:

- *Study Information*: Authors, year, journal, and geographical setting.
- *Surgical Details*: Approach (extraperitoneal or transperitoneal), patient cohort, surgeon experience.
- *Outcome Measures*: Operative time, blood loss, length of hospital stay, complication rates, oncologic margins, continence, erectile function recovery, and comparison with multiport or open techniques.
- *Technical Insights*: Learning curve, use of adjunct tools (e.g., EndoWrist SP), and instrument limitations.

The data extraction was conducted independently by two reviewers to ensure accuracy and consistency.

### Quality evaluation

The quality of included studies was appraised using validated tools suitable for the study design: Cochrane Risk of Bias Tool for RCTs, Newcastle-Ottawa Scale (NOS) for observational studies, and the AMSTAR 2 checklist for reviews. Methodological soundness, reporting clarity, and bias risk were assessed. Any low-quality or ambiguous studies were flagged and discussed, though none were excluded solely based on appraisal scores.

A summary table illustrating the quality scores and risk of bias assessments for the included studies were created and are included in the Results section. This provides a transparent overview of the evidence quality across different study types.

### Data synthesis

Due to the heterogeneity in study types, surgical techniques, and outcome measures, a qualitative narrative synthesis was adopted. Studies were grouped by thematic domains: surgical innovations, perioperative outcomes, functional and oncologic results, and comparative evaluations. This format allowed for a structured presentation of key insights while accounting for methodological diversity. A quantitative meta-analysis was not conducted due to substantial heterogeneity across the included studies in how perioperative and intraoperative outcomes were reported. Specifically, there was inconsistency in the statistical metrics used (e.g., means vs. medians, presence or absence of standard deviations), differences in follow-up intervals, and variability in surgical techniques and patient selection criteria. These factors limited the feasibility of valid data pooling. To preserve the integrity and comparability of the findings, a structured narrative synthesis was adopted instead, grouping results by outcome domains and study design.

A flowchart illustrating the study selection process is provided, summarizing the number of records identified, screened, and ultimately included in the review.

## Results

### Study characteristics

A total of 100 studies published between 2018 and 2025 were included in this review (Table 1). These studies encompassed a diverse range of research methodologies. Among them, 4 were randomized controlled trials evaluating the safety and efficacy of SP-RARP, 30 were observational studies reporting perioperative and functional outcomes in clinical practice, and 25 were systematic reviews synthesizing the current evidence base. Additionally, 10 meta-analyses provided pooled comparisons between SP and multiport approaches, while 31 narrative or technical articles explored surgical techniques, learning curves, instrumentation, and future applications of the da Vinci SP system.

The majority of studies were conducted in high-volume academic centers across North America, Europe, and East Asia. Both transperitoneal and extraperitoneal surgical approaches were represented, with the da Vinci SP platform serving as the unified technological focus of all included studies.

**Table 1:** Study characteristics of included articles

Study type	Number of studies	Key characteristics
Randomized Controlled Trials	4	Compared SP-RARP to MP-RARP in short-term functional outcomes
Observational Studies	30	Single- or multi-institution series reporting feasibility and outcomes
Systematic Reviews	25	Summarized functional, perioperative, and oncologic outcomes
Meta-analyses	10	Quantitative synthesis of SP vs. MP approaches
Narrative/Technical Reports	31	Focused on technique evolution, learning curves, and robotic innovations

### Major outcomes

Single-Port Robot-Assisted Radical Prostatectomy (SP-RARP) using the da Vinci SP system was associated with consistently favorable perioperative and functional outcomes across the reviewed literature. Comparative studies and institutional series underscored its feasibility and safety, with growing evidence supporting its non-inferiority to multi-port approaches (Table 2).

### Perioperative outcomes

SP-RARP demonstrated several perioperative advantages in high-volume centers and experienced hands. These included:

- Reduced estimated blood loss, with most studies reporting averages below 150 mL.
- Shorter hospital stays, often under 24 h in enhanced recovery settings.
- Lower postoperative pain scores, contributing to reduced opioid use.
- Improved cosmesis due to the single, umbilical incision.

Some observational studies noted slightly longer operative times during the initial learning phase; however, this effect diminished with increased surgeon experience, with times converging toward those of conventional multiport RARP [8, 19].

### Functional outcomes

Functional recovery, particularly with respect to urinary continence and erectile function, was comparable to multiport RARP in the short to intermediate term:

- Continence rates at 3 months ranged from 70 to 90% across multiple series.
- Erectile function preservation was more variable and often dependent on baseline function and nerve-sparing technique.

Several studies employed early continence-enhancing strategies such as posterior musculofascial reconstruction and anterior suspension sutures, with improved outcomes [33, 48].

### Oncologic outcomes

Short-term oncologic control was not compromised in SP-RARP:

- Positive surgical margin (PSM) rates ranged from 15 to 25%, aligning with multiport benchmarks.
- Biochemical recurrence (BCR) rates were low in available short-term follow-up studies, though long-term data remain sparse.

Meta-analyses and systematic reviews confirmed equivalent oncologic performance when compared to multiport RARP, especially for organ-confined disease [10, 40].

### Technical feasibility and learning curve

Initial experiences highlighted challenges unique to the da Vinci SP system, including:

- Limited instrument triangulation.
- Reduced assistant access.
- Steeper learning curve during the first 20–30 cases.

However, several adaptations—including extraperitoneal access, wristed SP instruments, and modular port configurations—were shown to mitigate these limitations over time [9, 47].

**Table 2:** Summary of major outcomes in SP-RARP studies

Outcome domain	Key findings	Supporting study types
Perioperative	Reduced blood loss Shorter hospital stay Lower pain and analgesia use Improved cosmesis	Observational studies, Meta-analyses
Functional	− 70–90% continence at 3 months Variable erectile function recovery Enhanced by reconstruction techniques	RCTs, Observational studies, Systematic reviews
Oncologic	PSM rates: 15–25% Low short-term BCR rates Comparable to MP-RARP	Meta-analyses, Systematic reviews, RCTs
Technical feasibility	Challenges: limited triangulation, instrument crowding Learning curve: 20–30 cases Innovations improve ergonomics	Narrative reports, Technical studies, Observational
Approach variation	Both transperitoneal and extraperitoneal used No clear superiority between them in outcomes	Observational studies, Technical comparisons

### Bias and quality assessment

A formal assessment of study quality was performed using tools appropriate to study design. The Cochrane Risk of Bias tool was used for RCTs, the Newcastle-Ottawa Scale for observational studies, and the AMSTAR 2 checklist for systematic reviews and meta-analyses (Table 3).

**Table 3:** Quality and bias assessment summary

Study type	Number of studies	Bias assessment tool	Overall risk of bias
Randomized controlled trials	4	Cochrane Risk of Bias	Low
Observational studies	30	Newcastle-Ottawa Scale (NOS)	Moderate
Systematic reviews	25	AMSTAR 2	Moderate
Meta-analyses	10	AMSTAR 2	Moderate
Narrative/Technical reports	31	Descriptive only – not graded	Not applicable

## Discussion

### Evolution of robotic surgery in urology

​The evolution of robotic surgery in urology has been marked by significant milestones, transitioning from open surgical techniques to advanced minimally invasive procedures. Initially, open radical prostatectomy was the standard approach for treating localized prostate cancer [9]. However, the advent of laparoscopic techniques in the late 20th century introduced less invasive options, albeit with limitations in instrument dexterity and visualization. These challenges paved the way for the development of robotic-assisted surgeries, offering enhanced precision and control [10].​

The introduction of the da Vinci Surgical System by Intuitive Surgical in 2000 revolutionized urologic surgeries. This system provided surgeons with a three-dimensional view and articulated instruments, improving surgical outcomes and reducing patient recovery times [11]. Over the years, the da Vinci system underwent several upgrades, including the Si and Xi models, which offered improved ergonomics and instrument flexibility [12]. These advancements solidified robotic-assisted radical prostatectomy (RARP) as a preferred method for prostate cancer treatment, with studies demonstrating its efficacy in preserving urinary and sexual functions compared to traditional approaches [13].​

Recognizing the need for even less invasive procedures, Intuitive Surgical developed the da Vinci Single-Port (SP) system. Launched in 2018, the SP system was designed to perform surgeries through a single incision, minimizing tissue trauma and enhancing cosmetic outcomes [14]. The system features a flexible camera and three multi-jointed instruments that can be deployed through a single cannula, allowing for complex procedures in confined spaces. This innovation has been particularly beneficial in urologic surgeries, where access to deep pelvic structures is required [15].​

The transition from multiport to single-port robotic platforms represents a significant advancement in surgical technology. Early adopters of the SP system have reported favorable outcomes, including reduced postoperative pain, shorter hospital stays, and quicker return to daily activities [14]. Moreover, the SP system’s design facilitates approaches such as the Retzius-sparing technique, which has been associated with improved urinary continence rates post-surgery. As the SP system continues to gain traction, ongoing studies aim to further validate its benefits and expand its applications within urology [16].

### The Da Vinci SP system: technical overview

​The da Vinci Single-Port (SP) Surgical System represents a significant advancement in robotic-assisted surgery, particularly within urology. Designed to perform complex procedures through a single incision, the SP system aims to minimize surgical trauma while maintaining the precision and control characteristic of robotic platforms [17].​

The SP system features a single 25 mm cannula that accommodates a fully wristed 3D HD endoscope and three multi-jointed, wristed instruments. This configuration allows for triangulation and a range of motion comparable to multiport systems, despite the single access point [18]. The instruments’ design facilitates intricate maneuvers within confined spaces, making the SP system particularly suited for pelvic surgeries such as radical prostatectomy. Additionally, the system’s compact setup and reduced number of external arms can lead to a less cluttered operating field and potentially shorter setup times [19].​

**Fig. 1:**
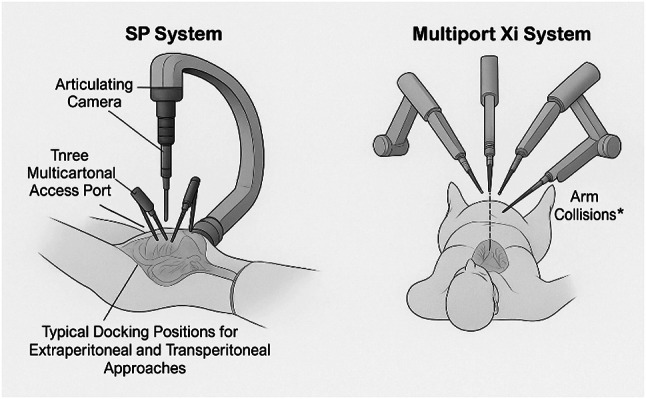
Configuration of the da Vinci Single-Port (SP) surgical system. The left panel illustrates the SP setup using a 25 mm multichannel access port inserted via an infraumbilical incision. It features a central articulating 3D HD endoscope and three wristed, multi-jointed instruments enabling intracorporeal triangulation. The diagram also shows typical docking sites for extraperitoneal and transperitoneal approaches. The right panel contrasts this with the multiport da Vinci Xi system, emphasizing its larger external footprint and increased risk of robotic arm collisions. Compared to Xi, the SP system offers a streamlined configuration that reduces operative clutter and improves access in confined pelvic spaces

When compared to earlier da Vinci models, such as the Si and Xi systems, the SP system offers distinct advantages and some limitations. The Si system, introduced in 2009, provided high-definition 3D visualization and improved ergonomics over its predecessors [20]. However, it required multiple ports and had bulkier arms, which could lead to external collisions and longer setup times. The Xi system (Fig. 1), released in 2014, addressed some of these issues with slimmer arms, an overhead boom for greater flexibility, and the ability to dock from any angle, enhancing its versatility for multi-quadrant surgeries [21]. Despite these improvements, both the Si and Xi systems still relied on multiple incisions, which could contribute to increased postoperative pain and longer recovery times [14].​

The SP system’s single-incision approach reduces the number of entry points, potentially decreasing postoperative discomfort and improving cosmetic outcomes. Its design also simplifies the docking process, as all instruments are introduced through a single port, reducing the time required for setup and minimizing the risk of arm collisions [22]. However, the SP system lacks certain features present in multiport systems, such as integrated suction and energy devices, necessitating the use of an assistant port or alternative techniques for these functions [23]. Additionally, the limited range of motion inherent to the single-port design can pose challenges in certain surgical scenarios, requiring surgeons to adapt their techniques accordingly [10].​

The transition to the SP system involves a learning curve, as surgeons must become proficient with the new instrument dynamics and visualization techniques. Studies have indicated that, while initial cases may take longer, operative times decrease as the surgical team gains experience with the system [24]. Furthermore, the SP system’s ergonomic design, including the intuitive control interface and improved instrument articulation, can reduce surgeon fatigue and enhance precision during procedures [25].​

In terms of setup and instrumentation, the SP system streamlines the process by utilizing a single port for all instruments, contrasting with the multiple ports required for the Si and Xi systems. This consolidation can lead to shorter docking times and a more efficient operating room workflow [26]. However, the need for specialized instruments and the current lack of certain integrated tools may limit the system’s applicability in some complex cases. Ongoing developments and adaptations are expected to address these limitations, expanding the SP system’s utility across a broader range of surgical procedures [27].

### Surgical technique of SP-RARP

​SP-RARP utilizing the da Vinci SP system represents a significant advancement in minimally invasive urologic oncology. This technique aims to reduce surgical morbidity while maintaining oncologic efficacy [28].​

Patient positioning is crucial for optimal access and visualization. In the extraperitoneal approach, patients are placed in a supine position with minimal Trendelenburg tilt, typically around 5–10°, which reduces the risk of cardiopulmonary complications associated with steep positioning [29]. A 3-cm infraumbilical incision is made to access the extraperitoneal space, which is then developed using blunt dissection or a balloon dilator. The SP access port is inserted through this incision, and the da Vinci SP system is docked accordingly [30].​

The surgical procedure begins with posterior dissection. The peritoneum is incised to expose the seminal vesicles and vas deferens, which are then dissected and mobilized. The camera is positioned at the 6-o’clock orientation during this phase to facilitate visualization [31]. Subsequently, the camera is repositioned to 12 o’clock to develop the Retzius space by transecting the median umbilical ligament, allowing for anterior bladder neck dissection. The bladder neck is then transected, and the prostate is dissected from the bladder. Neurovascular bundle preservation is performed when oncologically appropriate, followed by apical dissection and urethral transection [31, 32].​

Reconstruction involves posterior and anterior approaches to ensure a watertight vesicourethral anastomosis. Posterior reconstruction is achieved using a running suture to approximate the Denonvilliers’ fascia to the posterior bladder neck. Anterior reconstruction involves approximating the anterior bladder neck muscle fibers to the parietal pelvic fascia, which aids in hemostasis and supports early continence recovery [33, 34].​

The Retzius-sparing approach in SP-RARP has been explored to preserve continence mechanisms by avoiding dissection of the Retzius space. This technique involves a transvesical or posterior approach to the prostate, allowing for preservation of the anterior structures [35]. Early studies have demonstrated the feasibility of this approach with promising continence outcomes, although it requires advanced surgical expertise due to limited working space and altered anatomical landmarks [36, 37].​

The extraperitoneal approach offers several advantages, including reduced bowel manipulation, minimized risk of intraperitoneal complications, and suitability for patients with prior abdominal surgeries. This approach allows for direct access to the prostate without entering the peritoneal cavity, which can lead to faster recovery and decreased postoperative pain [38].

### Clinical outcomes

SP-RARP has emerged as a minimally invasive surgical option for prostate cancer, aiming to optimize oncologic control while enhancing functional recovery. Evaluating clinical outcomes such as margin status, lymph node yield, continence, and erectile function is crucial to assess the efficacy of this approach [39].​

Oncologic outcomes are pivotal in determining the success of SP-RARP. Positive surgical margin (PSM) rates serve as a critical indicator of oncologic control. A systematic review and pooled analysis reported a PSM rate of 33% across various studies, with higher rates observed in patients with advanced pathological stages (pT3) [40, 41]. Similarly, a single-center study noted a PSM rate of 35%, predominantly in patients with pT3 disease. These findings suggest that while SP-RARP is effective, the PSM rates are comparable to those reported in multiport RARP series, emphasizing the need for meticulous surgical technique, especially in locally advanced cases [42, 43].​

Lymph node yield during pelvic lymph node dissection (PLND) is another important oncologic parameter. In SP-RARP, the mean number of lymph nodes removed varies among studies. For instance, one study reported an average yield of 5.9 lymph nodes, with a subset of patients presenting with lymph node metastasis [44]. In contrast, another study documented a median of 19 lymph nodes removed, indicating variability that may be influenced by surgical approach and extent of PLND performed. These variations underscore the importance of standardized surgical protocols to ensure comprehensive oncologic assessment [45].​

Functional outcomes, particularly urinary continence and erectile function recovery, significantly impact postoperative quality of life. Continence rates following SP-RARP have shown promising results [46]. A study reported that 78% of patients achieved full continence at a median of 21 days postoperatively. Similarly, another study observed an 82.5% pad-free continence rate, highlighting the potential benefits of the single-port approach in facilitating early continence recovery [47, 48].​

Erectile function recovery post-SP-RARP varies, influenced by factors such as preoperative sexual health and nerve-sparing techniques. In a cohort where nerve-sparing procedures were performed, 64.4% of men with preoperative Sexual Health Inventory for Men (SHIM) scores above 20 regained potency [49]. Another study reported that 45.2% of patients recovered erectile function sufficient for intercourse within three months postoperatively. These outcomes suggest that SP-RARP can achieve satisfactory sexual function recovery, particularly when nerve-sparing techniques are employed [50].​.

Comparative analyses between SP-RARP and multiport RARP provide further insights into the efficacy of the single-port approach. A propensity score-matched study found no significant differences in PSM rates (19.4% in both groups) and three-month continence (77.4% vs. 83.9%) and potency rates (45.2% vs. 48.4%) between SP-RARP and multiport RARP [51–53]. These findings indicate that SP-RARP offers comparable oncologic and functional outcomes to the traditional multiport approach, with the added benefits of reduced invasiveness and potentially shorter recovery times.​

Early versus long-term outcomes are essential to comprehensively evaluate the benefits of SP-RARP. Short-term data demonstrate favorable results in terms of operative metrics and immediate postoperative recovery [54, 55]. However, long-term data, particularly regarding oncologic control and functional preservation, remain limited. Continued follow-up and larger cohort studies are necessary to substantiate the durability of these outcomes and to identify any late-onset complications or recurrences.​

In conclusion, SP-RARP demonstrates promising oncologic and functional outcomes in the short term, with PSM rates and functional recovery metrics comparable to those of multiport RARP. The technique’s minimally invasive nature may offer advantages in patient recovery and satisfaction. Nonetheless, further research with extended follow-up periods is warranted to fully establish the long-term efficacy and safety of SP-RARP in the management of prostate cancer.

### Perioperative considerations

SP-RARP has emerged as an innovative approach in the surgical management of prostate cancer, aiming to enhance perioperative outcomes while maintaining oncologic efficacy. Evaluating parameters such as operative time, estimated blood loss, length of hospital stay, intraoperative complications, conversion rates, postoperative pain, and cosmetic results is essential to comprehensively assess the benefits and limitations of this technique [56, 57].​

Operative time is a critical factor influencing surgical efficiency and patient recovery. Studies comparing SP-RARP to multiport RARP (MP-RARP) have reported comparable operative durations. For instance, a meta-analysis encompassing multiple studies found no significant difference in operative times between the two approaches, indicating that SP-RARP does not compromise surgical efficiency [52, 58]. Similarly, a single-institution study reported median operative times of 142.5 min for SP-RARP and 134.0 min for MP-RARP, with no statistically significant difference [59].​

Estimated blood loss (EBL) is another vital perioperative parameter. The aforementioned meta-analysis reported similar EBL between SP-RARP and MP-RARP. Additionally, a study observed median EBLs of 123.0 mL for SP-RARP and 120.0 mL for MP-RARP, indicating no significant difference [60]. These findings suggest that SP-RARP is comparable to MP-RARP concerning intraoperative blood loss.​

Length of hospital stay is an important indicator of postoperative recovery. Evidence suggests that SP-RARP may offer advantages in this regard. The meta-analysis noted a significant reduction in hospital stay for SP-RARP patients compared to those undergoing MP-RARP [61]. Furthermore, the single-institution study reported a median hospital stay of 4.5 days for SP-RARP patients versus 7.0 days for MP-RARP patients, highlighting a potential benefit of the single-port approach in facilitating earlier discharge [62].

Assessing intraoperative complications and the need for conversion to alternative surgical methods is crucial for evaluating the safety of SP-RARP. A comprehensive analysis from the Single-Port Advanced Research Consortium (SPARC) involving 1,103 patients undergoing SP-RARP reported a low intraoperative complication rate of 0.4% [63]. Notably, all intraoperative complications occurred during the transperitoneal approach and included bowel serosal tears, bladder injuries, and an obturator nerve injury. This suggests that while SP-RARP is generally safe, the choice of surgical approach may influence the risk of intraoperative complications [64].​.

Conversion rates from SP-RARP to MP-RARP or open surgery are also indicative of the procedure’s feasibility. The SPARC study did not report specific conversion rates, implying that conversions were infrequent. Similarly, other studies have demonstrated the technical feasibility of SP-RARP without significant need for conversion, reinforcing its viability as a surgical option [42, 65].​

Postoperative pain management is integral to patient satisfaction and recovery. Evidence indicates that SP-RARP may be associated with reduced postoperative pain compared to MP-RARP. A systematic review and meta-analysis reported that SP-RARP patients had a lower likelihood of requiring postoperative analgesia and opioids [51]. Furthermore, a study observed that SP-RARP patients had significantly lower pain scores at 8, 12, and 16 h postoperatively, suggesting enhanced patient comfort [66].​

Cosmetic outcomes, while subjective, are increasingly recognized as important factors in patient satisfaction. The single-port approach utilizes a single incision, typically concealed within the umbilicus, resulting in a virtually scarless appearance [67]. This contrasts with the multiple incisions required for MP-RARP, which may be more noticeable. Although direct comparative studies on cosmetic outcomes are limited, the inherent design of SP-RARP suggests a favorable cosmetic advantage [51].

### Comparative studies

Comparative analyses of SP-RARP versus multiport robot-assisted radical prostatectomy (MP-RARP), laparoscopic radical prostatectomy (LRP), and open radical prostatectomy (ORP) have become increasingly prevalent as surgical techniques evolve [68]. These studies aim to evaluate differences in perioperative, functional, and oncologic outcomes among these approaches.​

A systematic review and meta-analysis by Lv et al. (2023) compared SP-RARP and MP-RARP, encompassing seven studies with a total of 1,239 patients. The analysis revealed that SP-RARP was associated with a shorter hospital stay (weighted mean difference [WMD] − 17.86 h), reduced catheterization time (WMD − 1.51 days), and lower postoperative opioid use (odds ratio [OR] 0.26) compared to MP-RARP. Importantly, there were no significant differences in operative time, blood loss, continence and potency rates, complication rates, positive surgical margins, or biochemical recurrence between the two approaches [69].​

Further supporting these findings, a propensity score-matched analysis by Noh et al. (2022) compared SP-RARP and MP-RARP in 62 patients. The study found no significant differences in console time, operation time, estimated blood loss, positive surgical margins, or 3-month continence and potency rates between the two groups. However, SP-RARP was associated with lower nerve-sparing scores, suggesting a potential limitation in nerve preservation with the single-port approach [42].​

**Fig. 2:**
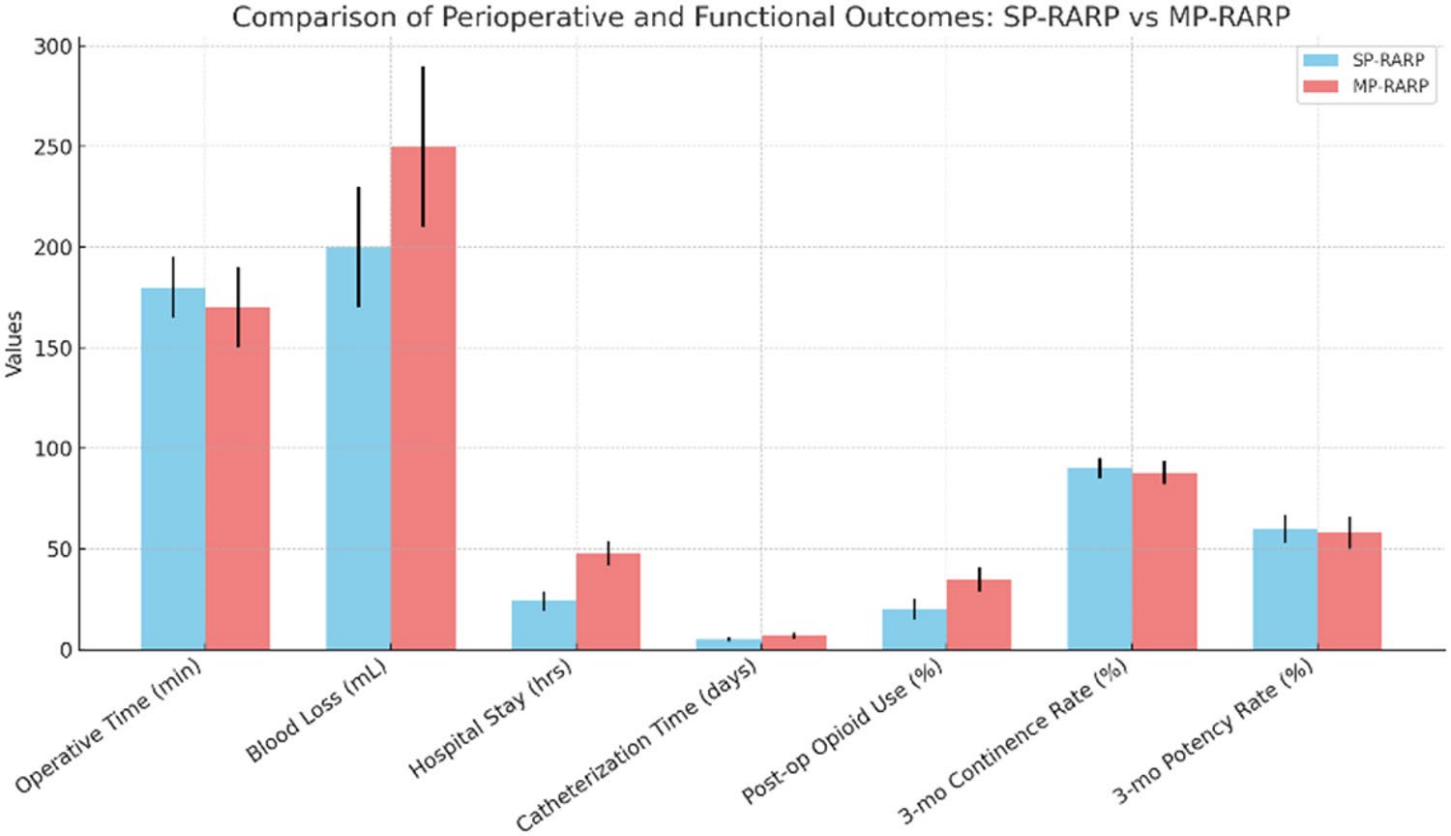
Bar graph comparing perioperative and functional outcomes of SP-RARP and MP-RARP. SP-RARP showed shorter hospital stays, reduced catheterization times, and lower postoperative opioid use, with similar operative times, blood loss, continence, and potency rates. Error bars indicate standard deviations

In a larger cohort study, Chavali et al. (2024) compared single-port extraperitoneal (SP EP) and multiport transperitoneal (MP TP) RARP in 606 patients. The SP EP approach demonstrated benefits such as reduced intraoperative blood loss (125.1 mL vs. 215.9 mL), shorter hospital stay (12.6 h vs. 31.9 h), decreased opioid use at discharge (14.4% vs. 85.1%), and earlier catheter removal (6 days vs. 8 days) (Fig. 2). Oncologic outcomes, including positive surgical margin rates and biochemical recurrence, as well as functional outcomes like continence and potency rates, were comparable between the two groups [52].​

When comparing robotic approaches to laparoscopic and open prostatectomy, a systematic review and network meta-analysis by Kim et al. (2025) evaluated 35 studies. The analysis found that robot-assisted radical prostatectomy (RARP) had lower biochemical recurrence rates compared to ORP (relative risk [RR] 0.713) and LRP (RR 0.672). RARP also had a significantly lower positive surgical margin rate compared to ORP (RR 0.893). Regarding functional outcomes, RARP showed higher potency rates than both ORP (RR 1.201) and LRP (RR 1.438), with no significant differences in continence rates among the three approaches [70]. Perioperative outcomes such as estimated blood loss and complication rates were similar across all surgical methods.​

In a study by Haese et al. (2019), involving 10,790 patients treated by highly trained surgeons, RARP and ORP were compared. The study found no significant differences in oncologic outcomes, including 48-month biochemical recurrence rates. Functional outcomes, such as 12-month continence and potency rates, were also similar between the two groups. However, RARP was associated with lower blood loss, transfusion rates, and shorter time to catheter removal, highlighting some perioperative advantages of the robotic approach [71].​

Overall, these comparative studies (Table 4) suggest that SP-RARP offers perioperative benefits such as reduced hospital stay, lower postoperative pain, and earlier catheter removal compared to MP-RARP, without compromising oncologic and functional outcomes. When compared to LRP and ORP, robotic approaches, including SP-RARP, demonstrate advantages in biochemical recurrence rates, positive surgical margins, and potency rates, while maintaining similar continence and complication rates. These findings support the continued adoption and refinement of robotic techniques in radical prostatectomy.

**Table 4:** Comparative outcomes of SP-RARP vs. MP-RARP, LRP, and ORP

Study	Comparison	Sample size	Key findings	Notable advantages of SP-RARP / RARP
Lv et al. 2023	SP-RARP vs. MP-RARP (Meta-analysis of 7 studies)	1,239	↓ Hospital stay (− 17.86 h) ↓ Catheterization time (− 1.51 days) ↓ Opioid use (OR 0.26) No difference in oncologic/functional outcomes	Shorter recovery time, less pain management needed
Noh et al. 2022	SP-RARP vs. MP-RARP (Propensity-matched)	62	No difference in console time, EBL, PSM, continence/potency ↓ Nerve-sparing scores in SP-RARP	Similar outcomes overall; possible limitations in nerve preservation
Chavali et al. 2024	SP EP-RARP vs. MP TP-RARP	606	↓ Blood loss (125.1 vs. 215.9 mL) ↓ Hospital stay (12.6 vs. 31.9 h) ↓ Opioid use (14.4% vs. 85.1%) ↓ Catheter time (6 vs. 8 days) Similar oncologic/functional outcomes	Enhanced recovery profile with SP extraperitoneal approach
Kim et al. 2025	RARP vs. ORP vs. LRP (Network Meta-analysis of 35 studies)	-	↓ Biochemical recurrence: RARP vs. ORP (RR 0.713), LRP (RR 0.672) ↓ PSM: RARP vs. ORP (RR 0.893) ↑ Potency: RARP vs. ORP (RR 1.201), LRP (RR 1.438) Continence rates similar	Superior oncologic and erectile function outcomes with RARP
Haese et al. 2019	RARP vs. ORP (High-volume surgeons)	10,790	No difference in 48-mo BCR, continence, or potency rates ↓ Blood loss, transfusions, catheter duration with RARP	Improved perioperative profile with equivalent long-term outcomes

*SP-RARP* single-port robotic-assisted radical prostatectomy, *MP-RARP* multi-port robotic-assisted radical prostatectomy, *EP* extraperitoneal, *TP* transperitoneal, *ORP* open radical prostatectomy, *LRP* laparoscopic radical prostatectomy, *EBL* estimated blood loss, *PSM* positive surgical margins, *BCR* biochemical recurrence, *RR* relative risk, *OR* odds ratio

### SP-RARP in practice: perspectives, patient experiences, and technological insights

Single-port robotic-assisted radical prostatectomy (SP-RARP) continues to gain traction in urological surgery due to its potential for reduced postoperative pain, shorter hospitalization, and improved cosmesis. This section integrates practical insights from surgical teams and highlights evolving technologies such as the EndoWrist SP that support this minimally invasive approach [72].

From the surgeon’s standpoint, transitioning to SP-RARP represents a notable shift in robotic technique. For instance, initial institutional experiences, such as those at Mayo Clinic, emphasized the promise of single-port laparoscopy to reduce procedural morbidity. However, early adoption was limited by ergonomic and technical challenges, including restricted triangulation and a steeper learning curve [73]. With structured training and growing procedural volume, many surgeons have reported decreased operative times and enhanced intraoperative control. The da Vinci SP system, with its 25-mm multichannel port and articulating camera system, has been credited with improving precision and workspace efficiency [22].

Clinical reports also suggest promising postoperative outcomes. For example, combined approaches using the da Vinci SP platform with adjunct visualization systems, such as Levita Magnetics’ MARS, have been implemented to optimize nerve-sparing techniques and improve functional preservation. These strategies aim to enhance erectile function and minimize postoperative discomfort—factors increasingly emphasized in quality-of-life assessments [74].

Technological advancements continue to play a pivotal role. The EndoWrist SP, with its double-jointed articulation, provides enhanced dexterity and minimizes instrument clashing within the confined operative field. This improved range of motion allows for more complex maneuvers and has been associated with smoother surgical execution and potentially improved outcomes [75].

Collectively, the practical integration of SP-RARP in clinical settings demonstrates both its feasibility and its potential advantages. While technical demands remain, ongoing innovations in surgical instrumentation and refinement of technique continue to support broader adoption of SP-RARP as a viable, minimally invasive alternative in prostate cancer surgery.

### Challenges and limitations

SP-RARP represents a significant advance in minimally invasive surgery, offering potential advantages in terms of cosmetic outcomes and postoperative recovery. However, like any novel technology, it faces several challenges and limitations that must be carefully considered. These challenges encompass aspects such as the learning curve, cost, instrument crowding, and the selection of appropriate patients for optimal surgical outcomes [76].

The learning curve is one of the most significant obstacles to the widespread adoption of SP-RARP. Compared to multiport robotic-assisted radical prostatectomy (MP-RARP), which benefits from established techniques and a more familiar instrument setup, SP-RARP requires surgeons to adapt to new ways of performing procedures through a single trocar [47]. This adaptation can be difficult, particularly when performing complex maneuvers that require precise instrument manipulation. Numerous studies highlight the increased difficulty and longer learning curve associated with SP-RARP, especially for surgeons transitioning from traditional or multiport techniques [77]. One study found that the learning curve for SP-RARP is particularly steep during the early stages, with surgeons requiring significant practice to become proficient in manipulating the robotic instruments within a constrained space [78]. Furthermore, surgeons accustomed to the expansive field of view offered by multiport platforms may find the narrower single-port view limiting, which can further complicate the learning process [79].

Beyond the learning curve, the training requirements for mastering SP-RARP are substantial. As with any robotic-assisted surgery, adequate simulation and hands-on practice are crucial to developing competence. A robust training program that includes both virtual simulation and supervised clinical experience is necessary for ensuring patient safety and optimal surgical outcomes [80]. However, access to comprehensive training programs is not always available, which may limit the ability of some surgeons or institutions to adopt SP-RARP. The lack of a standardized curriculum for training in single-port robotic surgery further exacerbates this issue, leading to disparities in proficiency across different surgical centers [81]. Additionally, because SP-RARP is a relatively new technique, many hospitals may not have the infrastructure or the number of procedures required to allow surgeons to reach the required level of expertise [82].

Another key limitation of SP-RARP is its cost and accessibility. Robotic surgery in general is an expensive undertaking, with costs associated not only with the purchase and maintenance of the robotic systems but also with training and infrastructure [83]. The SP platform, in particular, is still a relatively novel addition to robotic-assisted surgery, and its cost is often higher than that of traditional multiport systems. This can be a significant barrier for many healthcare institutions, particularly in regions with limited resources [84]. Moreover, insurance coverage for SP-RARP is not universally available, making it difficult for patients to access the procedure, particularly in countries or healthcare systems with strict cost-control measures [85]. The financial burden posed by the cost of robotic surgery may also influence the decision-making process for both patients and healthcare providers, with many opting for less expensive alternatives, such as laparoscopic or open prostatectomy.

In addition to the financial aspects, another notable limitation of SP-RARP is the issue of instrument crowding and limited triangulation. One of the key differences between SP-RARP and MP-RARP is the need to perform all surgical maneuvers through a single port [86]. This creates significant limitations in terms of instrument movement and positioning, as the surgical tools must be carefully arranged to prevent interference. Instrument crowding, where multiple instruments are positioned within a limited space, is a common challenge in SP-RARP. This can lead to difficulty in maintaining optimal angles and reach for precise dissection or suturing, making certain aspects of the surgery more challenging than in the multiport approach. Additionally, the restricted triangulation inherent in single-port surgery limits the freedom of movement of the robotic arms, which can affect the surgeon’s ability to perform certain maneuvers with the same ease and precision as in multiport robotic surgery [87]. This limitation is particularly problematic in cases requiring extensive dissection or when there is limited visibility of the surgical site.

Finally, patient selection is critical to the success of SP-RARP. Not all patients are ideal candidates for single-port surgery. For instance, patients with obesity or large abdomens may present significant challenges due to the limited space available for instrument maneuvering [88]. In such cases, the surgeon may find it difficult to achieve the necessary access and visibility for an optimal procedure. Similarly, patients with a history of significant pelvic surgery or those with abnormal anatomical features may not be suitable candidates for SP-RARP, as the single-port approach may not provide adequate exposure of critical structures [89]. Careful preoperative assessment is necessary to identify the best candidates for SP-RARP, and some surgeons may prefer to reserve this approach for specific types of prostate cancer or patient demographics.

Despite these challenges, the continued development of single-port robotic surgery and improvements in both technology and training programs may help mitigate many of these limitations. Further research is required to refine techniques and overcome the obstacles related to instrument crowding and triangulation, as well as to evaluate the long-term benefits and risks associated with the SP approach.

### Innovations and future directions

​Single-port robotic-assisted surgery (SP-RAS) has emerged as a significant advancement in minimally invasive procedures, offering potential benefits such as reduced postoperative pain, shorter hospital stays, and improved cosmetic outcomes. Looking ahead, several innovations and future directions are poised to further enhance the capabilities and applications of SP-RAS [90].​

The integration of augmented reality (AR) and artificial intelligence (AI) into SP platforms represents a promising frontier. AR can provide surgeons with enhanced visualization by overlaying critical anatomical structures onto the surgical field, thereby improving precision during procedures [91]. For instance, AI-assisted AR systems have been developed to create patient-specific 3D models from preoperative imaging, facilitating better planning and intraoperative navigation [92]. These technologies can assist in identifying anatomical landmarks and potential anomalies, leading to more informed decision-making and potentially improved outcomes. However, challenges such as system integration, real-time processing, and user training must be addressed to fully realize the benefits of AR and AI in SP-RAS [93].​

Beyond prostatectomy, SP-RAS is expanding its role in other urologic procedures, including nephrectomy and cystectomy. Early experiences with SP-RAS for partial nephrectomy have demonstrated its feasibility and safety, with studies reporting successful outcomes and a low rate of complications [94]. Similarly, the application of SP-RAS in radical cystectomy has shown promise, offering advantages such as reduced postoperative pain and shorter recovery times [95]. These procedures benefit from the minimally invasive nature of SP-RAS, which allows for precise tissue handling and minimal disruption to surrounding structures. As surgical techniques and technologies continue to evolve, the adoption of SP-RAS in these areas is expected to increase, potentially setting new standards for patient care [96].​

The establishment of multi-institutional trials and registries is crucial for evaluating the long-term outcomes and effectiveness of SP-RAS across diverse populations. Collaborative research efforts can provide robust data on patient selection criteria, procedural techniques, and postoperative care, contributing to the development of best practice guidelines [97]. For example, the creation of a national registry for robotic-assisted surgeries has facilitated the collection of comprehensive data, enabling comparisons between different surgical approaches and the identification of factors influencing patient outcomes [98]. Such initiatives are essential for building a strong evidence base to support the widespread adoption of SP-RAS.​.

The potential for outpatient or same-day discharge protocols following SP-RAS is an area of active exploration. Studies have demonstrated that patients undergoing SP-RAS often experience shorter hospital stays, with some being discharged on the same day as the surgery [99]. This trend is attributed to the minimally invasive nature of the procedure, which typically results in less postoperative pain and a quicker return to normal activities. Implementing same-day discharge protocols requires careful patient selection, comprehensive preoperative education, and coordinated postoperative care to ensure patient safety and satisfaction [100]. As healthcare systems strive to improve efficiency and reduce costs, the adoption of outpatient protocols for SP-RAS may become more widespread.​

## Conclusion

SP-RARP represents a promising advancement in minimally invasive urologic surgery, offering several clinical benefits, including reduced postoperative pain, faster recovery, and improved cosmetic outcomes. The da Vinci SP system, with its flexible instruments and enhanced visualization, has been shown to improve surgical precision while minimizing incisions and recovery time. As evidenced by both surgeon perspectives and patient experiences, SP-RARP has the potential to become a transformative approach in prostate cancer treatment. However, challenges related to the learning curve, instrument crowding, and cost need to be addressed for broader adoption. Clinical outcomes, such as oncologic and functional recovery, are encouraging but require further validation through large-scale, multi-institutional studies.

Looking ahead, future research should focus on optimizing the technical aspects of SP-RARP, particularly regarding instrument design, augmented reality integration, and AI-assisted surgical navigation. Additionally, long-term data on oncologic and functional outcomes will be essential to establish the true efficacy and safety of SP-RARP in comparison to other approaches like multiport robotic-assisted and open surgeries. The potential for outpatient or same-day discharge protocols is another area for future exploration, which could significantly enhance patient care and reduce healthcare costs. For urologic oncology practice, adopting SP-RARP may lead to improved surgical precision and patient satisfaction, but its widespread integration will depend on ongoing technological advancements, surgeon training, and evidence-based support.

## Data Availability

No datasets were generated or analysed during the current study. Data sharing not applicable to this article as no data-sets were generated or analyzed during the current study.
